# Clinical Role of Adjuvant Chemotherapy after Radical Hysterectomy for FIGO Stage IB-IIA Cervical Cancer: Comparison with Adjuvant RT/CCRT Using Inverse-Probability-of-Treatment Weighting

**DOI:** 10.1371/journal.pone.0132298

**Published:** 2015-07-15

**Authors:** Phill-Seung Jung, Dae-Yeon Kim, Shin-Wha Lee, Jeong-Yeol Park, Dae-Shik Suh, Jong-Hyeok Kim, Yong-Man Kim, Young-Tak Kim, Joo-Hyun Nam

**Affiliations:** Division of Gynecolgic Oncology, Department of Obstetrics and Gynecology, University of Ulsan, College of Medicine, Asan Medical Center, Seoul, Korea; Northwestern University Feinberg School of Medicine, UNITED STATES

## Abstract

**Objective:**

To evaluate the clinical role of adjuvant chemotherapy (AC) in FIGO stage IB-IIA cervical cancer patients.

**Study Design:**

A cohort of 262 patients with cervical cancer who received radical hysterectomy (RH) and adjuvant therapy at Asan Medical Center between 1992 and 2012 was enrolled. In this cohort, 85 patients received adjuvant chemotherapy (AC), and 177 received adjuvant radiotherapy or concurrent chemoradiation therapy (AR). Oncologic outcomes and adverse events in both treatment arms were compared using weighted Cox proportional hazards regression models with inverse-probability-of-treatment weighting (IPTW) to reduce the impact of treatment selection bias and potential confounding factors.

**Results:**

During a 46.8-month median follow-up duration, 39 patients (14.9%) had recurrences, and 18 patients (6.9%) died of disease. In multivariate analysis, the hazard ratio (HR) for recurrence and death was not significantly different in patients in either treatment arm (*p*=0.62 and 0.12, respectively). Also, after IPTW matching, the HR for recurrence did not significantly differ between the arms (HR 1.57, 95% CI 0.68-3.62, *p*=0.29). Similarly, disease-free survival and overall survival were not significantly different between the arms (*p*=0.47 and 0.13, respectively). In addition, patients with AC had a much lower prevalence of long-term complications (lymphedema: n=8 (9.4%) vs. 46 (26.0%), *p*=0.03; ureteral stricture: n=0 vs. 9 (6.2%), *p*=0.05).

**Conclusion:**

Patients with FIGO stage IB-IIA cervical cancer can benefit from AC after RH with fewer long-term complications and non-inferior therapeutic effect to AR. Chemotherapy may therefore be an alternative adjuvant treatment option for cervical cancer, particularly in younger patients.

## Introduction

Cervical cancer is decreasing in incidence due to more regular cytologic screening programs and the development of vaccinations but still remains the third most common gynecologic cancer worldwide [1]. In Korea, cervical cancer is the most commonly diagnosed gynecologic cancer with over 3,000 new cases diagnosed annually accounting for 4.5% of new female cancer cases [2]. The standard treatments for cervical cancer are radical hysterectomy (RH) with lymphadenectomy or primary concurrent chemoradiation therapy (CCRT) [3–5]. Regarding postoperative adjuvant treatment for cervical cancers of FIGO stage IB to IIA, several international clinical trials have found that adjuvant radiotherapy or CCRT (AR) improves the disease-free survival (DFS) and overall survival (OS) outcomes in patients with pathologic risk factors for recurrence [6–8]. Adjuvant radiotherapy is recommended when postoperative pathologic examinations reveal more than two intermediate risk factors for recurrence, such as negative lymph nodes with a bulky tumor [BT; tumor diameter>4 cm], deep stromal invasion (DSI) and/or lymphovascular space invasion (LVSI) [6,8]. Adjuvant CCRT is recommended when recurrence is highly likely based on pathologic evidence of a lymph node metastasis (LNM), a positive resection margin (RM), and/or parametrial invasion (PMI) [3].

A development of modern radiation techniques, such as new highly conformal external-beam and brachytherapy or intensity-modulated radiotherapy, have led to important reductions in recurrence and patient morbidity and mortality. However, patients who underwent pelvic radiation may still experience adverse effects and toxicity because of the anatomic locations, such as bladder, bowel, vagina and ovary [9,10]. Since up to 42% of women diagnosed with cervical cancer are younger than 45 [11], quite a few patients are hesitate to choose AR with concerns about unexpected adverse effects, which are usually irreversible during their longer life span [12]. For this reason, gynecologic oncologists are often asked about alternative options of adjuvant treatment by patients considering the quality of life after adjuvant treatment. Although there have been several studies comparing clinical outcomes between patients treated with AR or adjuvant chemotherapy (AC) after RH and lymphadenectomy [13–16], there is no consensus regarding the role of AC in the treatment of FIGO stage IB to IIA cervical cancers. In our current study, we demonstrate the clinical role of AC after RH in FIGO stage IB-IIA cervical cancer patients by assessing the oncologic outcomes of patients that underwent AR after RH. Baseline characteristics and pathologic risk factors in both of these treatment arms were matched using the propensity score (PS) and the inverse-probability-of-treatment weighting (IPTW) method. The patterns of recurrence and the adverse effects of each adjuvant treatment approach were also analyzed.

## Materials and Methods

### Patients

(The Informed consent is not given because all data were analyzed retrospectively and anonymously.) Following institutional review board approval (S2013-0587-0001), patients that were diagnosed with cervical cancer and received RH at Asan Medical Center between January 1992 and December 2012 were identified from an electronic database at our center, and their medical records were reviewed retrospectively. The eligibility criteria for patient inclusion in this study were as follows: a clinical diagnosis of FIGO stage IB-IIA lesions; a pathological confirmation of cervical cancer; an age of between 20–80 years at the time of surgery; laparoscopic or open radical hysterectomy (type 2 or 3) with a pelvic and/or para-aortic lymphadenectomy; and received AC. Patients whose cell type was small cell carcinoma or neuroendocrine carcinoma, who underwent neoadjuvant chemotherapy, or who had other coexisting malignancies were excluded. A total of 85 patients satisfied our eligibility criteria. Using the same eligibility criteria but excluding the adjuvant treatment approach, we also collected 177 patients who underwent AR after RH and compared the oncologic outcomes and adverse effects of each adjuvant treatment. The patterns of recurrence were also evaluated.

### Adjuvant treatment

Adjuvant treatment was started 3 to 4 weeks after surgery for patients whose pathologic examination results revealed more than two intermediate risk factors for disease recurrence, such as BT, DSI (≥1/2 thickness), or LVSI, or more than one high risk factor such as LNM, positive RM or PMI. The surgeon decided which adjuvant treatment (AC or AR) the patient would receive based on pathologic report, age and surgeon’s preference. The radiotherapy protocol we use at the Asan Medical Center is as follows: the four-field box technique (anterior-posterior, posterior-anterior, and two bilateral fields) is used for radiotherapy with a dose of 45–55 Gy (median, 50.4 Gy) in conventional 28 fractionations. The radiation field includes the tumor bed and the regional lymphatic tissues. Brachytherapy is performed in cases of positive or close vaginal resection margins. CCRT comprises the same dose of radiation with concurrent cisplatin-based chemotherapy at 40 mg/m^2^ and is administered to patients showing high risk factors for recurrence.

The chemotherapeutic agents used to treat the cervical cancer patients at our center are cisplatin and 5-fluorouracil (PF) or taxane and platinum-based chemoagent (TP). Thirty-five patients received PF between 1992 and 2002 (1000mg/m^2^ of 5-fluorouracil and 60mg/m^2^ of cisplatin) and 59 patients received TP between 2000 and 2012 (135mg/m^2^ of taxane and 75mg/m^2^ of cisplatin). Patients received at least three courses of chemotherapy at 3- or 4-week intervals beginning 3 to 4 weeks after surgery. Chemotherapy was delayed if the absolute neutrophil count was less than 1,000/μL or the platelet count was less than 90,000/μL. After the completion of adjuvant treatment, patients were evaluated at 3-month intervals for 2 years and every 6 months thereafter. Pelvic examinations were done with liquid-based cytology from a vaginal stump at every visit, and imaging studies such as computed tomography or positron emission tomography were performed every 6–12 months or as clinically indicated.

### Statistical methods

The baseline characteristics of the patients in both treatment arms were compared using the Kruskal-Wallis Test for continuous variables and a Chi-square test for categorical variables. Univariate and multivariate analysis of all variables were also done using a Cox proportional hazards (PH) model. To reduce the impact of treatment selection bias and potential confounding factors, we performed rigorous adjustments for significant differences in patient characteristics using weighted Cox PH regression models with inverse-probability-of-treatment weighting (IPTW). The propensity scores (PS) were estimated by multiple logistic regression analysis. To determine the PS values, all prespecified covariates were included in the full nonparsimonious models for AC versus AR treatments. The discrimination and calibration abilities of each PS model were assessed using the C statistic and the Hosmer-Lemeshow statistic. With this technique, weights for patients in the AR group were the inverse of (1-PS), and weights for patients in the AC arm were the inverse of the PS. Following PS matching of both arms, the Wilcoxon signed rank test was used for continuous variables, and the McNemar’s test or marginal homogeneity test was used for categorical variables. The Cox PH model was applied using PS-based matching with robust standard errors. The DFS and OS values were estimated using the Kaplan-Meier method. All reported *p* values in this study are 2 sided, and *p* values <0.05 were considered to be statistically significant. SAS software version 9.3 (SAS Institute, Inc., Cary, NC) was used to perform these statistical analyses.

## Results

### Baseline characteristics and comparisons (n = 262) (S1 File)

The baseline characteristics and comparisons of the patients who received AC or AR are summarized in Table 1. Of the 262 subjects in our study cohort who received both surgery and adjuvant treatment, 85 patients (32.4%) received AC and 177 patients (67.66%) received AR. Cisplain-based chemotherapy were administered to all patients in AR group except for two patients,who had elevated creatinine level higher than 2.0mg/dL. Those two patients had concurrent weekly carboplatin with a dosage of 2 area under the curve instead of weekly cisplatin.

**Table 1:** Baseline characteristics and comparison of the study patients in the AC and AR treatment arms.

Variables (median)	Value (range)	AC	AR	*P* *
		(*n* = 85, 32.4%)	(*n* = 177, 67.6%)	
Age (years)	47 (25–77)	44 (25–71)	48 (25–77)	0.03
BMI (kg/m2)	23.0 (15.8–36.7)	22.9 (15.8–35.8)	23.6 (16.2–36.7)	0.61
Level of initial SCC Ag (U/mL)	1.3 (0.5–48.4)	1.4 (0.5–34.6)	1.3 (0.5–48.4)	0.59
Pathologic tumor size (mm)	34 (5–110)	30 (6–85)	35 (5–110)	0.54
Ratio of DOI	0.75 (0.06–1.0)	0.76 (0.5–1.0)	0.9 (0.06–1.0)	<.01
Ratio of DOI≥0.5, *n* (%)	223 (85.1%)	63 (75.9%)	154 (87.5%)	0.02
PMI, *n* (%)	81 (30.9%)	8 (9.5%)	73 (41.2%)	<.01
LVSI, *n* (%)	145 (55.3%)	46 (54.1%)	99 (55.9%)	0.78
RMI, *n* (%)	10(3.8%)	1 (1.2%)	9 (5.1%)	0.17
LNM, *n* (%)	107 (40.8%)	27 (31.8%)	80 (45.7%)	0.03
Cell type, *n* (%)				
Squamous cell carcinoma	199 (76.0%)	61 (71.8%)	138 (78.0%)	0.75
Adenosquamous	16 (6.1%)	3 (3.5%)	13 (7.3%)	
Adenocarcinoma	47 (17.9%)	21 (24.7%)	26 (14.7%)	
Stage, *n* (%)				
Ib1	188 (71.8%)	67 (78.8%)	121 (68.4%)	0.20
Ib2	42 (16.0%)	11 (12.9%)	31 (17.5%)	
IIa	32(12.2%)	7 (8.2%)	25 (14.1%)	
Recur, *n* (%)	39 (14.9%)	11 (12.9%)	28 (15.8%)	0.40
local	20 (51.3%)	6 (54.5%)	10 (35.7%)	0.30
distant	19 (48.7%)	5 (45.5%)	18 (64.3%)	
Death, *n* (%)	18 (6.9%)	3 (3.5%)	14 (7.9%)	
DFS (months)	43.8 (4.0–224.6)	48.9 (4.2–224.6)	42.6 (4.0–188.3)	
OS (months)	46.8 (7.3–224.6)	49.9 (7.3–224.6)	44.8 (7.6–188.3)	

AC: adjuvant chemotherapy; AR: adjuvant radiotherapy or concurrent chemoradiation; BMI: body mass index; DOI: depth of invasion (depth of tumor/cervical thickness); PMI: parametrial invasion; LVSI: lymphovascular space invasion; RMI: resection margin ivasion; LNM: lymph node metastasis; DFS: Disease-free survival; OS: overall survival.

*p-values were calculated using the Kruskal-Wallis Test for continuous variables and a Chi-square test of the Fisher's exact test for categorical variables.

During a 46.8-month median follow-up period (range: 7.3–224.6), 39 cases (14.9%) had recurrences and 18 patients (6.9%) died of cervical cancer. The median age of the patients in the AC arm was significantly less than the AR group; 44 (25–71) *vs*. 48 (25–77), respectively (*p* = 0.03). Patients in the AR arm appeared to have a significantly deeper DOI ratio (0.90 vs. 0.76, *p*<0.01), more PMI (41.2% vs. 9.5%, *p*<0.01), and more LNM (45.7% vs. 31.8%, *p*<0.01).

**Fig 1:**
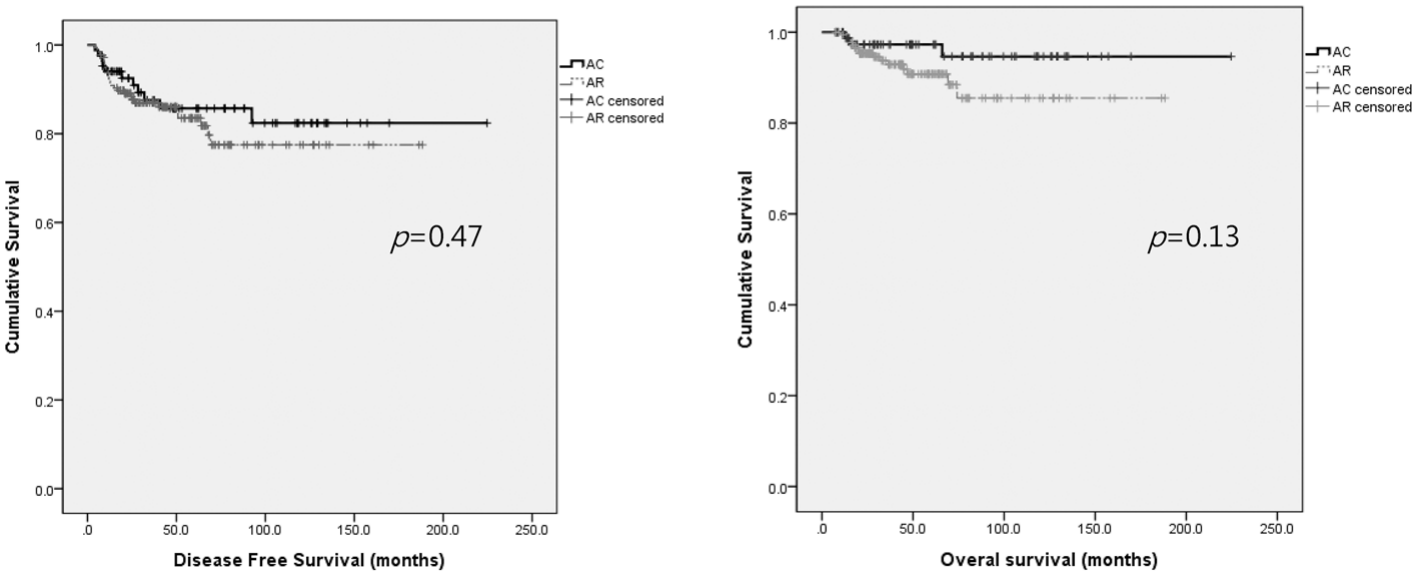
Kaplan-Meier survival curves indicating no significant differences in the progression-free survival or overall survival outcomes between patients who received adjuvant chemotherapy (AC, n = 85) and those who received adjuvant radiotherapy or concurrent chemoradiation therapy (AR, n = 177) (*p* = 0.47 and 0.13, respectively). AC: adjuvant chemotherapy; AR: adjuvant radiotherapy of concurrent chemoradiation.

As shown in Table 2, multivariate analysis revealed that the HR for recurrence was not significantly different between the two treatment arms (*p* = 0.62) and that PMI and LNM significantly impacted the recurrence rates (PMI HR 2.58 (95% CI 1.26–5.31, *p* = 0.01)), LNM HR 1.92 (95% CI 1.01–3.68, *p* = 0.04), respectively). Death was also not affected by adjuvant treatment (*p* = 0.12), and only LNM significantly affected the death rate (HR 2.94 (95% CI 1.07–7.85, *p* = 0.04)). The DFS and OS did not significantly differ between the arms (*p* = 0.47 and 0.13, respectively), as shown in Fig 1.

**Table 2:** Univariate and multivariate analysis of recurrence and death.

			Univariate analysis^a^				Multivariate analysis^b^			
		Recur (*n* = 39)	95% CI				95% CI			
Recurrence		*n*	HR	lower	upper	*p*	HR	lower	upper	*p*
AC		11	1				1			
AR		28	1.26	0.62	2.54	0.52	0.82	0.37	1.8	0.62
Pathologic tumor size (mm)			0.99	0.97	1.01	0.46	0.98	0.96	1.01	0.19
PMI	no	21	1				1			
	yes	18	2.28	1.215	4.31	0.01	2.58	1.26	5.31	0.01
LVSI	no	13	1							
	yes	26	1.66	0.85	3.22	0.14				
Ratio of DOI	<0.5	8	1				1			
	≥0.5	31	0.91	0.39	2.06	0.81	1.04	0.43	2.51	0.92
RMI	no	36	1							
	yes	3	2.29	0.7	7.48	0.17				
LNM	no	17	1				1			
	yes	22	2.03	1.08	3.81	0.02	1.92	1.01	3.68	0.04
			Univariate analysis^a^				Multivariate analysis*			
		Death (*n* = 18)	95% CI				95% CI			
Death		*n*	HR	lower	upper	*p*	HR	lower	upper	*p*
AC		3	1				1			
AR		15	2.56	0.73	8.93	0.14	2.73	0.76	9.79	0.12
Pathologic tumor size (mm)			1	0.98	1.03	0.81	1.01	0.98	1.04	0.66
PMI	no	9	1							
	yes	9	2.76	1.01	7.03	0.03				
LVSI	no	3	1							
	yes	15	4.1	1.19	14.15	0.03				
Ratio of DOI	<0.5	3	1				1			
	≥0.5	14	1.05	0.3	3.65	0.94	0.83	0.23	3.02	0.78
RMI	no	18								
	yes	0	.	.	.	0.99^''^				
LNM	no	6	1				1			
	yes	12	3.21	1.21	8.57	0.02	2.94	1.07	7.85	0.04

AC: adjuvant chemotherapy; AR: adjuvant radiotherapy or concurrent chemoradiation; PMI: parametrial invasion; LVSI: lymphovascular space invasion; DOI: depth of invasion (depth of tumor/cervical thickness); RMI: resection margin invasion; LNM: lymph node metastasis; CI: confidence interval; HR: hazard ratio.

^a^,^b^Cox Proportional Hazards model.

^''^Fisher's exact test.

### Subgroup analysis of patients with nodal metastasis (n = 107)

The baseline characteristics and comparisons of patients with a nodal metastasis are summarized in Table 3. In this subgroup, 27 patients (25.2%) received AC while 80 patients (74.8%) underwent AR. During the follow-up duration, 22 patients (20.6%) had a recurrence and 12 (11.2%) died of the cancer. Regarding risk factors for recurrence, larger tumor size (33 vs. 37mm, *p* = 0.02), deeper DOI ratio (0.70 vs. 0.87, *p* = 0.01), and PMI (11.1% vs. 43.8%, *p*<0.01) were found at significantly higher levels in the AR group.

**Table 3:** Baseline characteristics and comparison of patients in the AC and AR treatment arms from the nodal metastasis subgroup (*n* = 107).

Variables (median)	Value (range)	AC (*n* = 27, 25.2%)	AR (*n* = 80, 74.8%)	*p*
Age (years)	43 (25–71)	46 (25–71)	44 (27–66)	0.91
BMI (kg/m^2^)	23 (17.6–31.2)	22.3 (17.6–31.2)	23 (17.6–25.4)	0.49
Level of initial SCC Ag (U/mL)	2.0 (0.5–48.4)	1.3 (0.5–34.6)	2.2 (0.5–48.4)	0.48
Pathologic tumor size (mm)	35 (6–85)	33 (6–85)	37 (8–70)	0.02
Ratio of DOI^\^	0.9 (0.08–1.0)	0.70 (0.1–1.0)	0.87 (0.08–1.0)	0.01
Ratio of DOI≥0.5, *n* (%)	88 (82.2%)	17 (63.0%)	68 (86.1%)	0.04
PMI, *n* (%)	38 (35.5%)	3 (11.1%)	35 (43.8%)	<0.01
LVSI, *n* (%)	71 (66.4%)	16 (59.3%)	55 (68.8%)	0.37
RMI, *n* (%)	5 (4.7%)	0	5 (6.3%)	0.33
Stage, *n* (%)				
Ib1	80 (74.6%)	24 (88.9%)	56 (70.0%)	0.15
Ib2	18 (16.8%)	2 (7.4%)	16 (20.0%)	
IIa	9 (8.4%)	1 (3.7%)	8 (10.0%)	
Recur, *n* (%)	22 (20.6%)	6 (22.2%)	16 (20.0%)	0.50
local	7 (31.8%)	2 (7.4%)	5 (6.3%)	0.50
distant	15(65.2%)	4 (14.8%)	11 (13.2%)	
Death, *n* (%)	12 (11.2%)	2 (7.4%)	10 (12.5%)	
DFS (months)	39.7 (5.4–224.6)	56.0 (5.9–224.6)	42.5 (5.4–160.6)	
OS (months)	42.2 (7.5–227.9)	59.6 (7.5–227.9)	42.9 (7.6–160.6)	

AC: adjuvant chemotherapy; AR: adjuvant radiotherapy or concurrent chemoradiation; BMI: body mass index; DOI: depth of invasion (depth of tumor/cervical thickness); PMI: parametrial invasion; LVSI: lymphovascular space invasion; RMI: resection margin ivasion; DFS: Disease-free survival; OS: overall survival.

*p-values were calculated using the Kruskal-Wallis Test for continuous variables and a Chi-square test of the Fisher's exact test for categorical variables.

**Fig 2:**
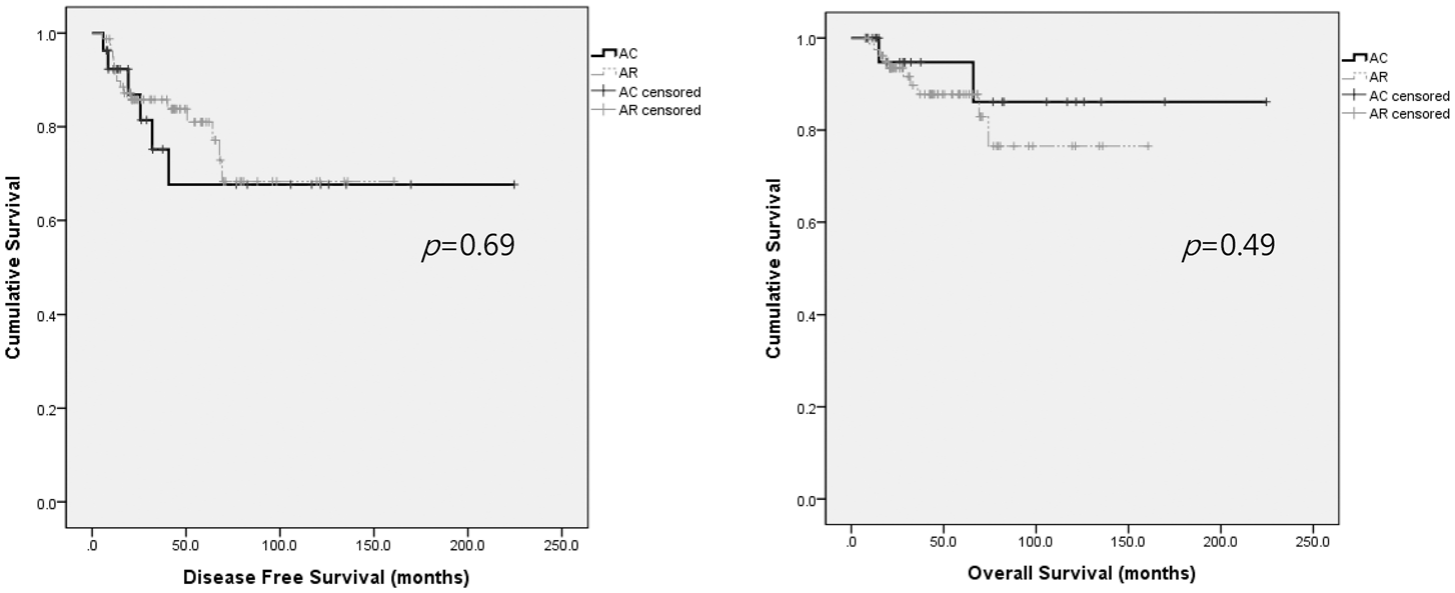
In the nodal metastasis subgroup, Kaplan-Meier survival curves also indicated no significant differences in progression-free survival or overall survival between patients radiotherapy or concurrent chemoradiation therapy (AR, n = 80) (*p* = 0.69 and 0.49, respectively). AC: adjuvant chemotherapy; AR: adjuvant radiotherapy of concurrent chemoradiation.

By multivariate analysis (Table 4), the adjuvant treatment did not significantly affect recurrence (*p* = 0.21). Only the PMI was found to be significantly associated with recurrence (HR 4.06, 95% CI 1.40–11.79, *p* = 0.01). None of the risk factors for recurrence or the adjuvant treatment were significantly associated with death. The DFS and OS of patients with nodal metastasis also did not significantly differ between the arms (*p* = 0.69 and 0.49, respectively), as shown in Fig 2.

**Table 4:** Univariate and multivariate analysis of recurrence and death in patients with a nodal metastasis.

			Univariate analysis^a^				Multivariable analysis^b^			
			95% CI				95% CI			
Recur (*n* = 22)		Number of patients	HR	lower	upper	*p*	HR	lower	upper	*p*
AC		6	1				1			
AR		16	0.81	0.32	2.01	0.66	0.48	0.15	1.52	0.21
Pathologic tumor size (mm)			0.99	0.96	1.02	0.62	0.98	0.94	1.01	0.22
PMI	no	10	1				1			
	yes	12	2.49	1.07	5.76	0.03	4.06	1.4	11.79	0.01
LVSI	no	5	1							
	yes	17	1.73	0.64	4.68	0.28				
Ratio of DOI	<0.5	5	1				1			
	≥0.5	17	0.82	0.3	2.26	0.71	1.12	0.36	3.53	0.84
RMI	no	20	1							
	yes	2	2.3	0.53	9.95	0.27				
			Univariate analysis^a^				Multivariable analysis^b^			
			95% CI				95% CI			
Death (*n* = 12)		Number of patients	HR	lower	upper	*p*	HR	lower	upper	*p*
AC		2	1				1			
AR		10	1.72	0.37	7.99	0.49	1.49	0.3	7.33	0.63
Pathologic tumor size (mm)			1.02	0.98	1.06	0.43	1.01	0.97	1.06	0.61
PMI	no	5	1							
	yes	7	2.28	0.88	8.78	0.08				
LVSI	no	2	1							
	yes	10	2.34	0.51	10.7	0.27				
Ratio of DOI	<0.5	3					1			
	≥0.5	9	1.34	0.29	6.26	0.71	1.14	0.21	6.12	0.88
RMI	no	12	1.33	0.29	6.2	0.72				
	yes	0	.	.	.	>0.99''				

AC: adjuvant chemotherapy; AR: adjuvant radiotherapy or concurrent chemoradiation; PMI: parametrial invasion; LVSI: lymphovascular space invasion; DOI: depth of invasion (depth of tumor/cervical thickness); RMI: resection margin ivasion; CI: confidence interval; HR: hazard ratio.

^a^,^b^ Cox Proportional Hazards model.

^''^Fisher's exact test.

### Comparison of recurrence between both treatment arms using the IPTW method

Using the IPTW method (Table 5), patients in the AC and AR treatment arms showed no significant differences in recurrence rate (HR 1.57 95% CI 0.68–3.62, *p* = 0.29). Regarding the subgroup with nodal metastasis, no significant differences were evident either between the AC and AR treatment arms in terms of the recurrence rate (HR 0.87 95% CI 0.23–3.28, *p* = 0.83).

**Table 5:** Comparison of recurrence between the AC and AR treatment arms by the Cox proportional hazard model using the inverse-probability-of-treatment weighting method.

All patients	HR	95% CI		*p*
AC	1			
AR	1.57	0.68	3.62	0.29
**Patients with nodal metastasis**				
AC	1			
AR	0.87	0.23	3.28	0.83

AC: adjuvant chemotherapy; AR: adjuvant radiotherapy or concurrent chemoradiation; HR: hazard ratio; CI: confidence interval.

### Patterns of recurrence and salvage treatment

**Fig 3:**
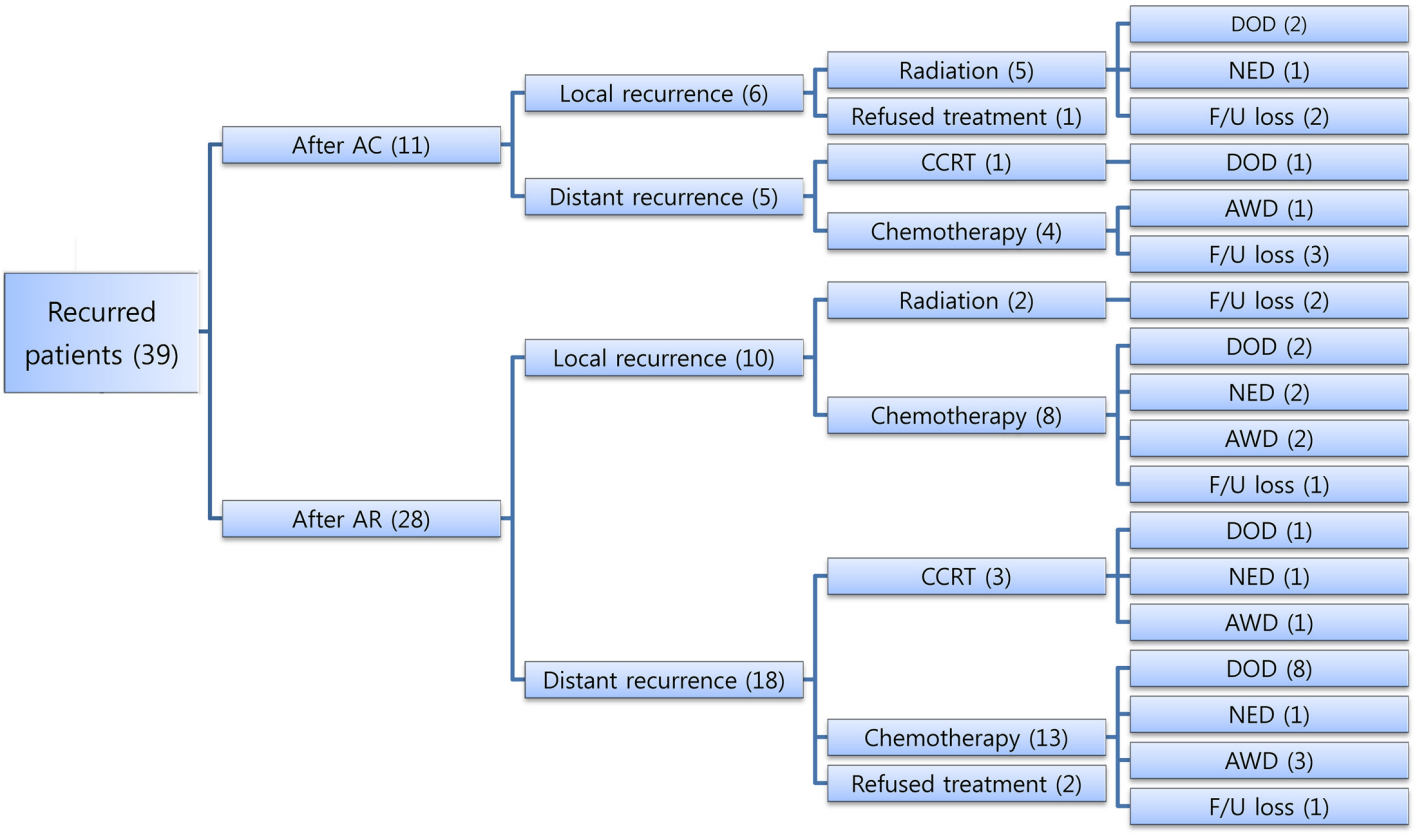
Salvage treatment and final status of the recurred patients (n = 39). AC: adjuvant chemotherapy; AR: adjuvant radiotherapy of concurrent chemoradiation; CCRT: concurrent chemoradiation; DOD: die of disease; NED: no evidence of disease; F/U: follow-up; AWD: alive with disease.

Thirty-nine patients developed recurrent disease after adjuvant treatment; 11 recurred after AC and 28 recurred after AR (Fig 3). Among the 11 AC patients with recurrences, 6 had local recurrence and 5 had systemic recurrence. Among individuals with a systemic recurrence after AC, 4 underwent systemic chemotherapy and 1 patient received CCRT. In the 6 cases of local recurrence after AC, 5 patients underwent CCRT. The remaining patient elected not to receive further treatment. Among the 28 patients with a recurrence after AR, 10 (35.7%) developed local recurrence and 18 (64.3%) showed a systemic recurrence. Eight of the patients with local recurrence and 13 with systemic recurrence underwent systemic chemotherapy. Two locally recurrent cases received radiotherapy and 3 patients with systemic recurrence underwent CCRT.

### Adverse effects of adjuvant treatment

Incidences of adverse effects during or after adjuvant treatment in our patient groups are indicated in Table 6. Among the 85 cervical cancer patients in the AC arm, 19 (22.4%) had complications, such as lower-limb lymphedema, small bowel ileus, peripheral neuropathy and acute hematologic complications. Among the 177 patients in the AR treatment arm, 67 (37.9%) had complications, including lower-limb lymphedema, small bowel ileus, ureteral stricture, rectovaginal fistula (RVF), radiation-related proctitis or cystitis, or vaginal stricture. Some of these patients had more than one complication simultaneously. The incidence of lymphedema was significantly higher in AR patients (26.0% *vs*. 9.4%, *p* = 0.03). Stricture of the ureter only occurred in AR patients (n = 9 (6.2%), *p* = 0.05), which necessitated a ureteral intervention such as double J catheterization or percutaneous nephrostomy. RVF also occurred only in AR patients (n = 3 (1.7%), *p* = 0.67) but this did not show statistical significance due to its low incidence. Acute hematologic complications following adjuvant treatment showed a significantly higher incidence in AC patients (9.4% *vs*. 0.5%, *p*<0.01).

**Table 6:** Adverse effects of adjuvant treatment.

Adjuvant treatment	Number of patients	Lymphedema	Ileus	Hematologic complications	Ureteral stricture	RVF	Radiation-related proctitis/cystitis	Vaginal dryness or stricture	Peripheral neuropathy
AC (*n* = 85)	19 (22.4%)	8 (9.4%)	2 (2.4%)	8 (9.4%)	0	0	0	0	1 (1.2%)
AR(*n* = 177)	67 (37.9%)	46 (26.0%)	7 (4.0%)	1 (0.5%)	9 (6.2%)	3 (1.7%)	6 (3.4%)	3 (1.7%)	0
^a^AC vs. AR		*p* = 0.03	*p* = 0.82	*p*<0.01	*p* = 0.05	*p* = 0.67	*p* = 0.23	*p* = 0.48	*p* = 0.61

AC: adjuvant chemotherapy; AR: adjuvant radiotherapy or concurrent chemoradiation; RVF: rectovaginal fistula.

^a^Fisher's exact test.

^b^all cases needed surgical correction.

^c^all cases needed ureteral intervention.

## Discussion

In our present observational study, we compared the clinical outcomes of FIGO stage IB-IIA cervical cancer patients who received AC or AR treatment after RH and lymphadenectomy using the IPTW method. Because the various risk factors for recurrence in cervical cancer make statistical analysis difficult, there have not been many previous studies comparing the treatment outcomes of AC and AR. In our current retrospective analyses, we enrolled all cervical cancer patients treated at our hospital with either intermediate or high risk factors for recurrence. To adjust confounding factors between two treatment arms, we used the IPTW method based on PS, which allowed us to design an observational study that mimics some of the particular characteristics of a randomized controlled trial. The IPTW method uses the weights based on the PS to create a synthetic sample in which the distribution of measured baseline covariates is independent of the treatment used [17,18]. With this statistical strength, we succeeded in showing that AC is not inferior to AR in treating cervical cancer, and is in fact superior to AR in terms of quality of life, as shown by a much lower incidence of long-term complications.

We also analyzed the patterns of recurrence in our study cohort. Due to the small number of recurrent patients, we did not find any significant difference in recurrence between the AC and AR arms. However, it can be assumed that AR is vulnerable to systemic coverage (64.3% *vs*. 45.5%, *p* = 0.30). Moreover, when choosing a salvage treatment option after recurrence, AC provides more options than AR because of reserved radiotherapy, especially for locally recurrent cases.

There have been several studies demonstrating equivalent therapeutic effects of AC after RH and lymphadenectomy in early cervical cancer cases. Takeshima et al. [19] reported a potential role of AC alone by demonstrating a similar recurrence rate and lower incidence of complications in patients with both intermediate- and high-risk factors. Although other studies have shown the clinical efficacy of AC in early cervical cancer, these reports were limited to patients with intermediate risk factors for recurrence, and the comparisons were made between chemotherapy and radiotherapy. Lee et al. [13] concluded that AC had a therapeutic efficacy for local control that was equivalent to radiotherapy, such that intrapelvic recurrence might be adequately prevented. Hosaka et al. [14] reported that AC did not worsen disease-free survival outcomes by reducing distant control, and that AC was superior in terms of postoperative complications following radiotherapy. In another report two years later [16], these same authors highlighted that AC might be more beneficial for survival than adjuvant radiotherapy.

There were some limitations to our current study of note. First of all, some patients were received AC, despite the current standard treatment with AR after RH for cervical cancer with risk factors for recurrence. However, it was not decided either accidentally or unreasonably. Thorough discussions with the multidisciplinary oncologic team at our center led us to choose AC as an adjuvant treatment option in some patients for several reasons. First, AC is considered to be more effective against hidden LNM because AR is insufficient for systemic coverage and the dosage of chemoagent is limited when co-administered with radiation. Second, for young patients who wanted to preserve their ovarian function or sexual activity, AC was preferred. Third, upon recurrence, patients with previous AC can choose either chemotherapy or CCRT as a salvage treatment, while patients who have received AR have fewer treatment options, which is thought to be due to radio-resistance.

Additionally, we could not demonstrate the HR for death because only a small number of patients died of their disease (n = 18, 6.9%). However, as shown in Figs 1 and 2, there were no significant differences evident between the treatment arms by Kaplan-Meier survival analysis, suggesting that AC is not inferior to AR. We also could not present the appropriate survival rate after salvage treatment because more than 25% of the recurred patients in our present cohort chose not to receive further treatment or were lost to follow-up. However, there are some studies that have reported a relatively high survival rate after local recurrence in patients treated with AC [14,20].

As we conducted a retrospective review of a 20-year period, the chemo-regimens used for patients in our AC arm were not homogeneous. Before 2002, PF was the standard combination of chemotherapeutic agents used for cervical cancer. Between 2000 and 2002, this was gradually replaced by TP. On the other hand, as our present analyses constituted a long-term follow-up at a single institution, the surgical techniques and radical approach to the use of RH to treat the study patients can be considered homogeneous.

According to the 2010 SEER data, the median age of diagnosis of cervical cancer is 49, and this disease tends to be more aggressive in younger women, even in early stage cases that might require adjuvant treatment after RH and lymphadenectomy. Radiation-induced complications are difficult to treat and, consequently, are related to a poorer quality of life. For young cervical cancer patients, particularly nulliparas, chemotherapy is often chosen before and sometimes after radical trachelectomy to preserve fertility [21–23]. Young patients with cervical cancer still want to retain their sexual and ovarian function, even after sacrificing fertility to RH. The current study is not to provide an evidence for substituting AR to AC after RH for cervical cancer, but to demonstrate that AC could lead a non-inferior therapeutic effect to AR through robust retrospective review of large data. Although prospective randomized clinical trials are still required, we could cautiously imply that chemotherapy might be a feasible adjuvant treatment option in young cervical cancer patients who desire the better preservation of their sexual and ovarian functions.

## Supporting Information

S1 File Minimal data set of the study cohort. (XLSX)
